# Third Harmonic Generation microscopy distinguishes malignant cell grade in human breast tissue biopsies

**DOI:** 10.1038/s41598-020-67857-y

**Published:** 2020-07-06

**Authors:** Evangelia Gavgiotaki, George Filippidis, Vassilis Tsafas, Savvas Bovasianos, George Kenanakis, Vasilios Georgoulias, Maria Tzardi, Sofia Agelaki, Irene Athanassakis

**Affiliations:** 1 Institute of Electronic Structure and Laser, Foundation for Research and Technology, 70013 Heraklion, Crete Greece; 2 Medical School, University of Crete, 70013 Heraklion, Crete Greece; 3 Department of Physics, University of Crete, 70013 Heraklion, Crete Greece; 4 Department of Biology, University of Crete, 70013 Heraklion, Crete Greece

**Keywords:** Biophysics, Cancer, Oncology, Optics and photonics

## Abstract

The ability to distinguish and grade malignant cells during surgical procedures in a fast, non-invasive and staining-free manner is of high importance in tumor management. To this extend, Third Harmonic Generation (THG), Second Harmonic Generation (SHG) and Fourier-Transform Infrared (FTIR) spectroscopy were applied to discriminate malignant from healthy cells in human breast tissue biopsies. Indeed, integration of non-linear processes into a single, unified microscopy platform offered complementary structural information within individual cells at the submicron level. Using a single laser beam, label-free THG imaging techniques provided important morphological information as to the mean nuclear and cytoplasmic area, cell volume and tissue intensity, which upon quantification could not only distinguish cancerous from benign breast tissues but also define disease severity. Simultaneously, collagen fibers that could be detected by SHG imaging showed a well structured continuity in benign tumor tissues, which were gradually disoriented along with disease severity. Combination of THG imaging with FTIR spectroscopy could provide a clearer distinction among the different grades of breast cancer, since FTIR analysis showed increased lipid concentrations in malignant tissues. Thus, the use of non-linear optical microscopy can be considered as powerful and harmless tool for tumor cell diagnostics even during real time surgery procedures.

## Introduction

Breast cancer is one of the most common malignancies in the female population worldwide, caused by a combination of genetic and environmental factors, while displaying different phenotypes according to the pathophysiology of each patient^1^. Breast cancer encompasses a heterogeneous array of tumor cell types that are classified according to their histological and molecular characteristics into at least four subtypes, each one associated with a different prognosis and course of treatment. Although researchers are trying to find universal markers to characterize each type of cancer, such as hormone receptors, HER2 and Ki67, inter-patient heterogeneity undermines current standards of practice with histological markers to accurately diagnose the grade of disease for all breast cancer patients (e.g., not equal levels of expression for a given marker between two patients where the underlying grade of their cancers is the same). However, in spite of marker expression, cancer cells share common features in the context of activation, increase or energetic pools, dynamic nuclear activity, which could be universally applied for diagnosis. If such features could present themselves for a given grade and preserved across all patients, they should be investigated for their utility to consistently diagnose grade. To this extend, novel non-invasive methods of non-linear optical microscopy were employed to investigate these features in different grades of human breast cancer biopsies and provide fast decision-making therapeutic strategies.

Label-free non-linear optical microscopy produces high-resolution images with rich functional and structural information based on the intrinsic contrast. It also provides increased sensitivity, which could potentially serve as an imaging method for the early detection of various types of cancer and inflammatory diseases^2–10^. Recently, the application of this technology has been proposed in clinical studies, while it has also been used for intravital imaging in biological models^11,12^. Until now, non-linear optical modalities, such as Multiphoton Excitation Fluorescence (MPEF), Second Harmonic Generation (SHG) and Third Harmonic Generation (THG) have been applied to detect responses to neoadjuvant therapies for cancer^13–15^ and to define surgical margins in tissues^10,13,16^. Commonly to other non-linear imaging techniques, THG not only enables recording of label-free images without causing photobleaching and photoxicity effects on the biological samples, but it also allows quantitative analysis^5–7,17,18^. Of note, THG signal can be enhanced by the presence of multilayered structures detected in membranes, lipid bodies^19^ and inhomogeneities.

Although various studies have shown the potential of label free non-linear imaging for cancer research, this is the first time that THG microscopy was employed for accurate imaging and signal quantification in breast cancer tissues. Indeed, at the cellular level THG signal has been largely correlated to lipid bodies (LBs)^6,19^, which are highly dynamic and functional cellular organelles, actively involved in inflammation and cancer^20^. Ιt is generally believed that cancer cells display metabolic reprogramming as compared to healthy cells, related not only to mechanisms of ATP synthesis through glycolysis, but also to those associated in the de novo synthesis of lipids. Under physiological conditions, normal/healthy cells maintain the lipid levels under control by regulating their uptake, synthesis, and mobilization from internal storages. In contrast, tumor cells tend to uptake larger amounts of lipids and enhance lipogenesis and carbohydrate production, while also increasing fatty acid β-oxidation^20–22^, mainly due to their extensive metabolic needs.

In the present study, THG and other non-linear optical modalities were utilized to shed light on the above features and examine their potential use in diagnosis for differentiating malignant from benign breast tissue samples and discriminating among the different grades of cancer. In order to allow further characterization of the tissue samples, the obtained non-linear data were also combined with FTIR spectroscopy measurements^23,24^. FTIR spectroscopy has been recognized as a powerful tool for the study of biological molecules including lipids, proteins, peptides, biomembranes and nucleic acids^23,24^. Within the last decade, this technique has also been used in the study of more complex systems, of tissues and clinical samples, including breast cancer biopsies^25–30^. Overall, the findings presented herein suggest that non-linear optical imaging techniques could define the tumor, evaluate the grade and the malignant or benign nature of the outgrowth, while FTIR spectra analysis could provide valuable complementary chemical information.

## Results

In an effort to examine whether THG imaging could distinguish malignant from healthy cells within breast tissues and provide information as to the severity of tumor invasion, paraffin embedded sections were submitted to qualitative and quantitative non-linear imaging as well as FTIR analysis.

**Figure 1:**
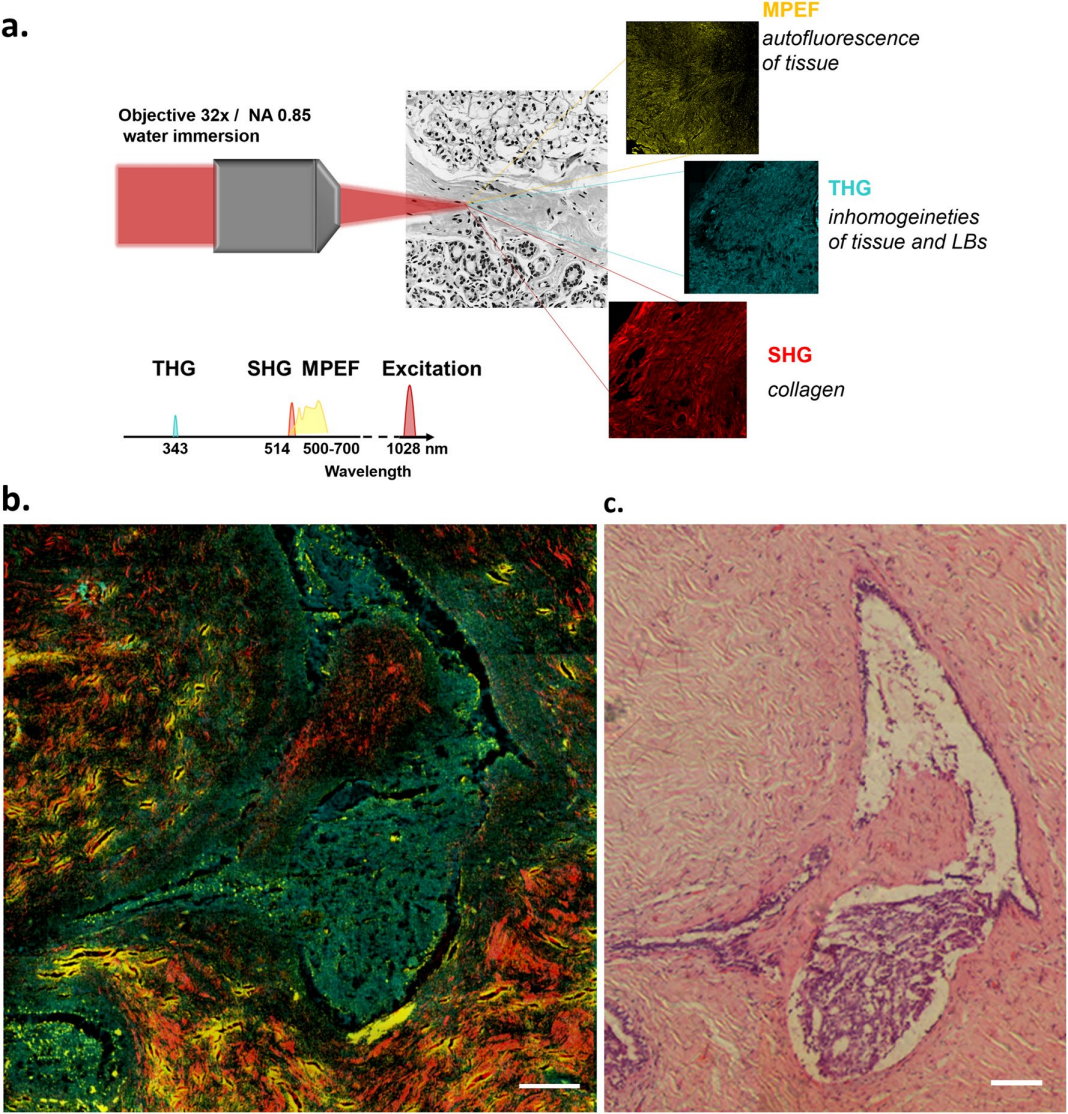
Schematic diagram for the collection of multimodal non-linear signal from breast tissue (**a**). Characteristic multimodal non-linear image (MPEF, THG, SHG) (**b**). THG signal is represented in cyan, SHG signal in red and MPEF in yellow color. The overlap of MPEF (yellow) and THG (cyan) signals generates green color, **c**) Corresponding H&E image. Scale bar depicts 100 μm.

Breast tissues from benign (grade 0) and grades I, II and III malignant tumors were submitted to multimodal non-linear imaging. The applied technology integrates non-linear processes, such as multiphoton fluorescence, second- and third-harmonic generation (SHG and THG), into a single, unified microscopy platform, providing thus complementary structural information within individual cells at the submicron level. Using a single beam, without the need of staining, information on autofluorescence (MPEF), inhomogeneities (mainly membranes and LBs, THG), as well as collagen distribution (SHG) could be visualized (Fig. 1a). Indeed, as shown in Fig. 1b, in a typical cancer tissue autofluorescence (yellow), inhomogeneities (cyan) and collagen (red) were easily distinguished and comparable to H&E staining (Fig. 1c). In the system described herein, autofluorescence corresponds to the combination of 2-photon and 3-photon excitation procedures, where NADH, elastin fibers and FAD represent the main contributors of the recorded MPEF signal^31,32^. NADH and elastin fibers present absorption maxima in the ultraviolet (UV) and FAD in the blue zone of the spectrum, while they all provide fluorescence signals in the visible. The use of a wavelength of 1,028 nm, allows 3-photon excitation for the detection of NADH and elastin fibers, and 2-photon excitation for the detection of flavins. Inhomogeneities, including membranes and LBs could be detected by THG imaging. It should be noted that in some paraffin-embedded protocols LBs are not retained by the fixation process. In this work, the applied methodology, which included osmium tetroxide/formalin-fixed tissues^33^ (see “Methods”) did not affect the LB content of the tissues (Supplement Fig. 1A). Because of the complexity of tissue sections, another parameter that had to be taken into consideration was whether empty spaces (holes) within the tissue, could be misinterpreted as THG signal. Indeed, such discrepancy could not occur, since THG signal was definitely acquired from cellular structures and not tissue empty spaces (Supplement Fig. 1B). Moreover, the use of such technology could detect the cell nucleus as black hole areas (no THG signal), since nuclear homogeneity makes THG signal to achieve a destructive interference^18^.

**Figure 2:**
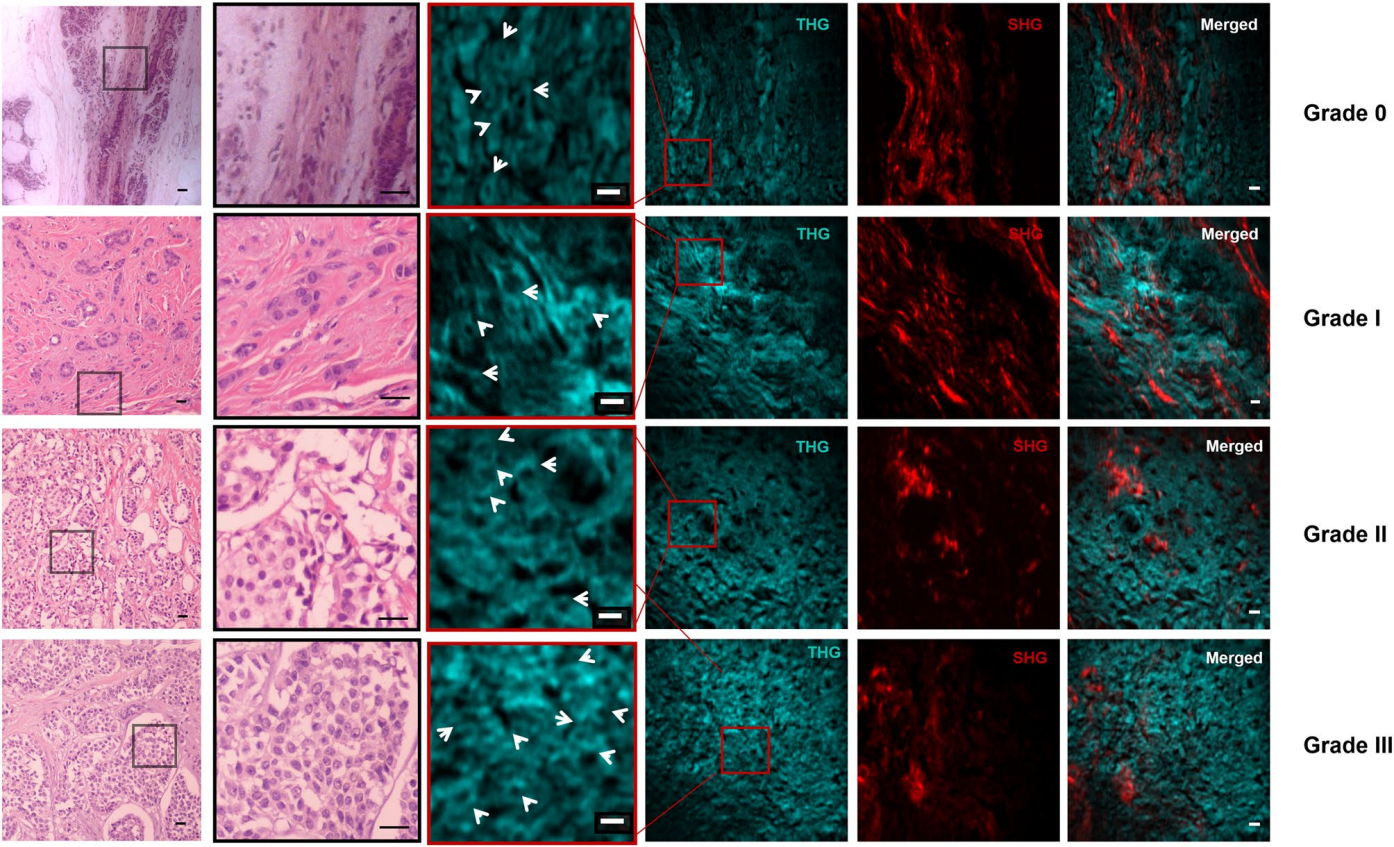
Hematoxylin and eosin (H&E) images and the corresponding non-linear images (SHG & THG) of benign (grade 0) and malignant (grade I, grade II, grade III) breast tissues. Characteristic THG images (cyan) of tissues show intracellular inhomogeneities, while SHG images (red) indicate collagen (scanning area ~ 150 μm^2^). Black squares in the H&E images indicate the regions that were scanned during the non-linear imaging measurements. The second column presents the zoomed H&E images. Red squares in the THG images represent the area that is enlarged in the third column (scanning area ~ 45 × 45μm^2^) to identify the cells. White arrows indicate typical cancer cells in the tissues. Scale bar depicts 5 μm for non-linear images and 10 μm for H&E images.

The described setup offers the opportunity for scanning region areas at the order of ~ mm^2^ of the tissue, depicting thus efficiently the malignant areas (Fig. 2). THG signal clearly showed differential organization in intracellular inhomogeneities, which become more compact from benign (grade 0) to grades I, II and III malignant tumors. At the same time, SHG signal that detects collagen fibers, showed that although in grade 0 tissues collagen orientation was well structured with specific continuity, in grades I, II and III collagen gradually lost its continuity and became less and less apparent (Fig. 2)^34–36^. However, MPEF signal did not show any significant changes with cancer progression (data not shown).

**Figure 3:**
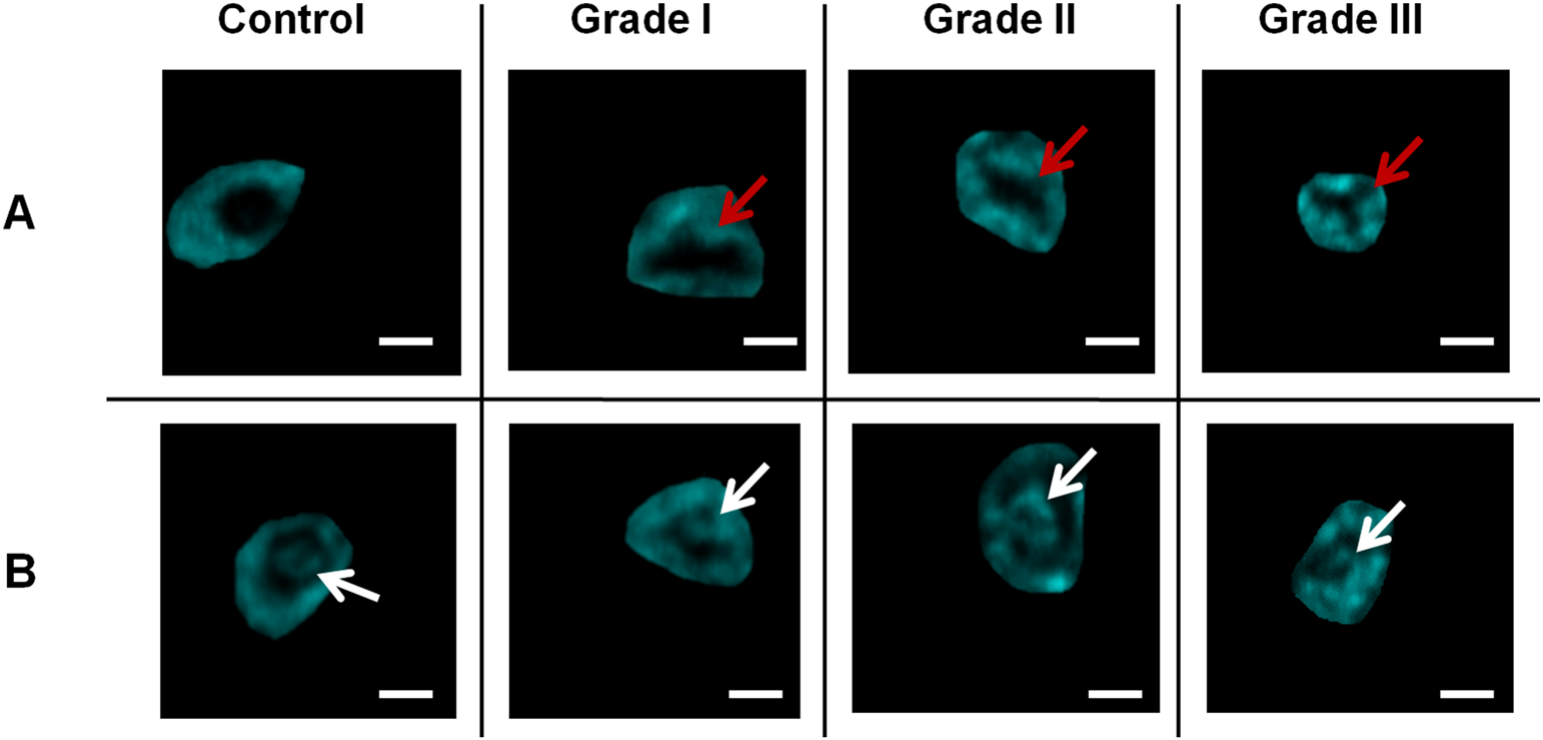
THG images of representative cells (from benign tissue left column and grades I, II and III of cancer at the following columns) showing irregularities in the nucleus (**A**) and nucleoli (**B**). Red arrows depict the irregular nucleus (shown as black area) in (**A**) and white arrows indicate nucleoli located within the nuclear area (interface of nucleus and nucleoli) in (**B**). Scale bar depicts 2 μm.

Because of the heterogeneous structure of breast tissue, the efforts to predict the behavior of the different lesions are limited to cytomorphology and biological marker analysis. Irregularities in both nuclear shape and size (‘pleomorphism’), coupled with changes in chromatin amount and distribution, remain the basic microscopic criteria for a cytologic diagnosis of cancer^37^. Morphologically, tumor cells are characterized by large nuclei of irregular size and shape, prominent nucleoli and reduced cytoplasm areas. The nucleus in neoplastic cells plays a key role in the assessment of tumor malignancy. Changes concerning the surface area, volume, the nucleus/cytoplasm (N/C) ratio, shape and density, as well as structure and homogeneity, serve as criteria in malignancy identification, features that could be easily detected by non-linear imaging without the need of staining^38^. To this extent, THG imaging analysis could discriminate and isolate specific cells within the tissues showing nuclear irregularities (Fig. 3A) and nucleoli inhomogeneities (Fig. 3B).

Further analysis concentrated on the morphometric characteristics of cells within benign and malignant tissues, which could also define the grade of the different cancerous cases^39,40^. Based on the recorded THG signal, the cells could be detected by the black central area (nucleus) and the surrounding inhomogeneities (membranes), isolated and submitted to quantification analysis. Such analysis included calculations of the mean nuclear area (MNA), mean cytoplasmic area (MCA) as well as the nuclear to cytoplasmic area ratio (N/C ratio) of cells (Table 1). It was interesting to note that MNA and MCA in grade 0 samples were higher as compared to grades I, II and III, while the N/C ratio was lower. In general, irregular nuclei showed increased N/C ratio, while conspicuous single prominent nucleoli could be detected in cancerous tissues. Cells in lower grade specimens showed moderate variation in nuclear size and shape, whereas higher grade specimens showed a variation in nuclear size and shape with a high N/C ratio (Table 1). Thus, a statistically significant increase of 39% (p < 0.0001), 54% (p < 0.0001) and 74% (p < 0.0001) of N/C ratio in grades I, II and III, was observed, respectively, when compared to grade 0 (Table 1).

**Table 1:** Tumor cell characteristics in tissue sections via THG imaging.

Grade	MNA ± SEM (μm^2^)	MCA ± SEM (μm^2^)	N/C ± SEM
0	82.54 ± 0.67	267.47 ± 0.67	0.254 ± 0.005
I	58.82 ± 0.47	161.38 ± 1.30	0.352 ± 0.003
II	77.35 ± 1.51	186.62 ± 0.62	0.392 ± 0.002
III	64.11 ± 0.52	145.32 ± 1.17	0.443 ± 0.002

N = 150 cells for each grade.

N/C ratio of cancerous tissues (Grade I–III) appear to have statistically significant difference (p < 0.0001****) respectively as compared to Grade 0 tissues.

*MNA* mean nuclear area, *MCA* mean cytoplasmic area, *N/C ratio* mean nuclear to cytoplasmic ratio.

**Figure 4:**
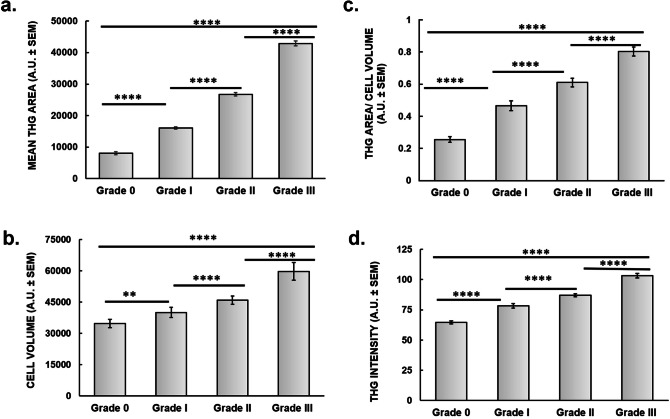
Quantification of non-linear measurements. Quantification of THG area of breast tissues (**a**) Quantification of cell volume (**b**) Quantification of mean THG area divided with cell volume (**c**) Quantification THG signal intensity (**d**). (N = 15 patients). N = 150 cells were tested from each category. For the statistical analysis (****) indicates p < 0.0001, (***) p < 0.001, (**) p < 0.01 and (*) p < 0.05.

Except from the morphological evaluation, THG modality also enabled quantitative analysis providing further information related to the cell area, cell volume as well as the THG signal intensity of a tissue area. After setting a constant threshold, cell detection and isolation was separately handled (see “Methods”). In each case, approximately 40 areas of 45 × 45 μm^2^ were evaluated for the analysis of N = 150 cells in grades 0–III cancerous areas of breast biopsies. THG signal area that represented the amount inhomogeneities of cells in the tissue areas tested was shown to increase with cancer severity (Fig. 4a). Thus, an increase of 99% (p < 0.0001), 231% (p < 0.0001) and 430% (p < 0.0001) of THG signal area in grades I, II and III specimens as compared to control (grade 0) areas was observed, respectively.

As described in methods, during THG imaging, the 2D optical sections combined to the scanning depth allowed cell volume calculations. Such experimentation showed that cancer cells displayed a statistically significant increase of cell volume as compared to grade 0-derived cells, also correlating with cancer severity (p < 0.01, p < 0.0001 and p < 0.0001 for grades I, II and III respectively; Fig. 4b). Normalization of the obtained results by dividing THG signal area of each cell to the respective cell volume, reproduced the observed difference between benign and tumor cells in the tissues tested (p < 0.0001, Fig. 4c). Such analysis indicated that independently of the cell volume, a significant increase of the THG signal area could be obtained in malignant cells, which could also detect differences among grades 0, I, II and III.

In another type of analysis, THG signal intensity was evaluated in different tissue areas. In this case, quantification was also performed by setting a constant threshold (see “Methods”). Thus, THG signal intensity was shown to increase with cancer severity by 21% (p < 0.0001), 35% (p < 0.0001) and 60% (p < 0.0001) in grades I, II and III as compared to grade 0, respectively (Fig. 4d). Thus, Fig. 4 demonstrates unambiguously that the quantification of THG signals, arising from breast tissue discontinuities, can correlate the increased inhomogeneity with the higher grades of cancer severity.

**Figure 5:**
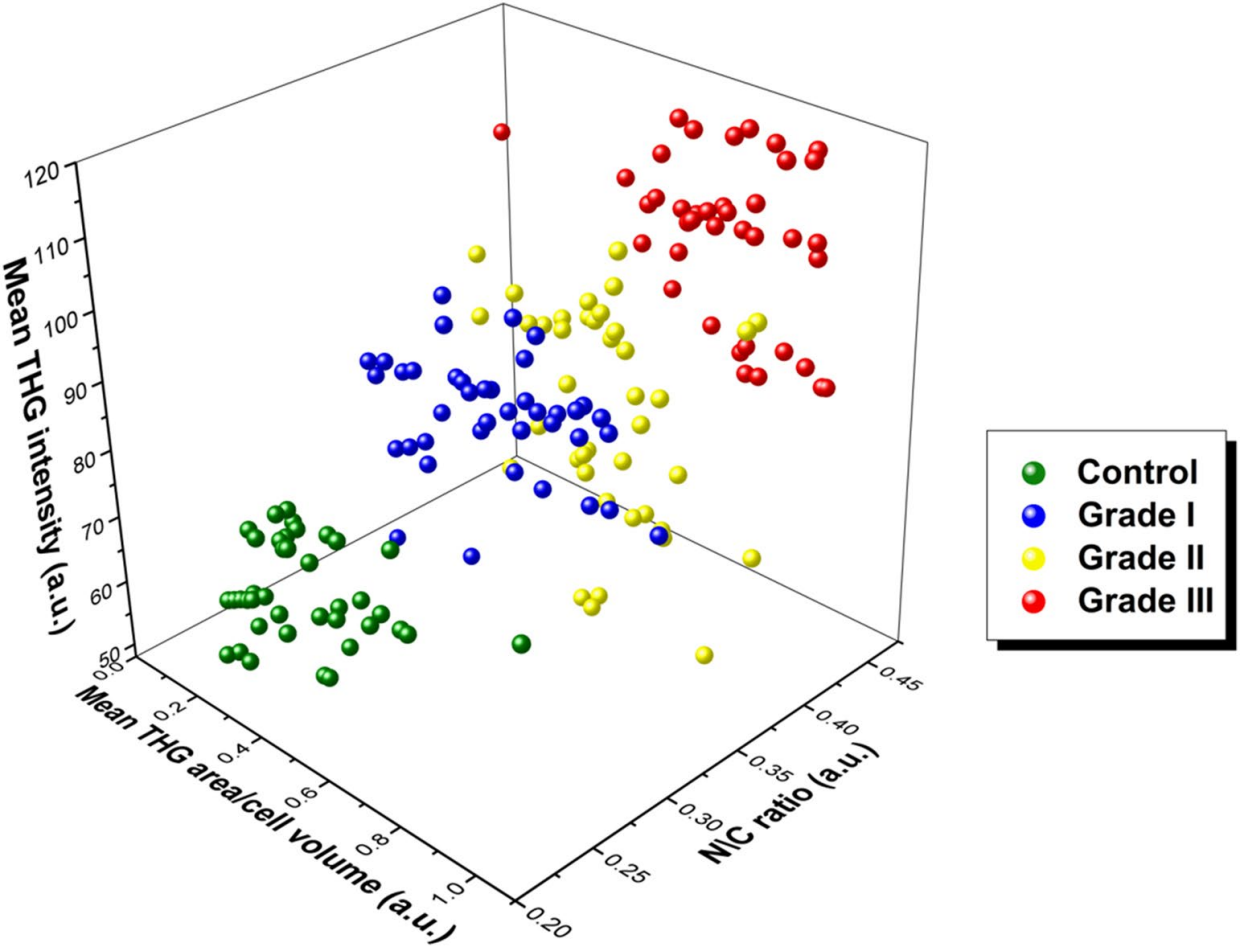
3D graph for the distinction between cancerous and control (grade 0) tissues via THG signals quantification. Mean THG area/cell volume area in x-axis, N/C ratio in y- axis and THG intensity values in z-axis. The grade III and the control tissues clearly appeared as separable sub populations of spots in the scatter plot (N = 40 for each tissue type).

Combining the results of the mean THG signal area/volume, N/C ratio for a representative number of N = 40 cells and THG tissue intensity, an indisputable discrimination of the different grades of malignant samples could be detected (Fig. 5).

Although higher grades of cancerous tissues were showing increased mean THG area, cell volume and N/C ratio, it was important to evaluate the absolute number of cells per surface area in the different cases tested. To this extend, cells with cancerous morphology, namely tubular differentiation, nuclear pliomorphism, irregular shape, non-homogeneous structures, could be distinguished from healthy control cells which coordinate with neighboring cells, show regular shape and small nucleus. For this type of analysis, the number of cells within areas of 0.002 mm^2^ was evaluated in each grade of malignant specimens. Thus, it was shown that 7 ± 1 cells could be detected in grade 0 specimens, 13 ± 1 cells in grade I, 21 ± 1 cells in grade II and 40 ± 1 cells in grade III tissues. Taking into consideration the mean THG signal area and cell volume of grade 0 cells, the percentage of healthy control cells within the cancerous tissues could be evaluated. Thus, in a 0.1 mm^2^ area, grade I samples included 39% of control cells, grade II 24%, while grade III 23% of control cells. Therefore, these results implied that the greater the tumor grade, the greater the number of cancerous cells, and the lesser number of control cells.

**Figure 6:**
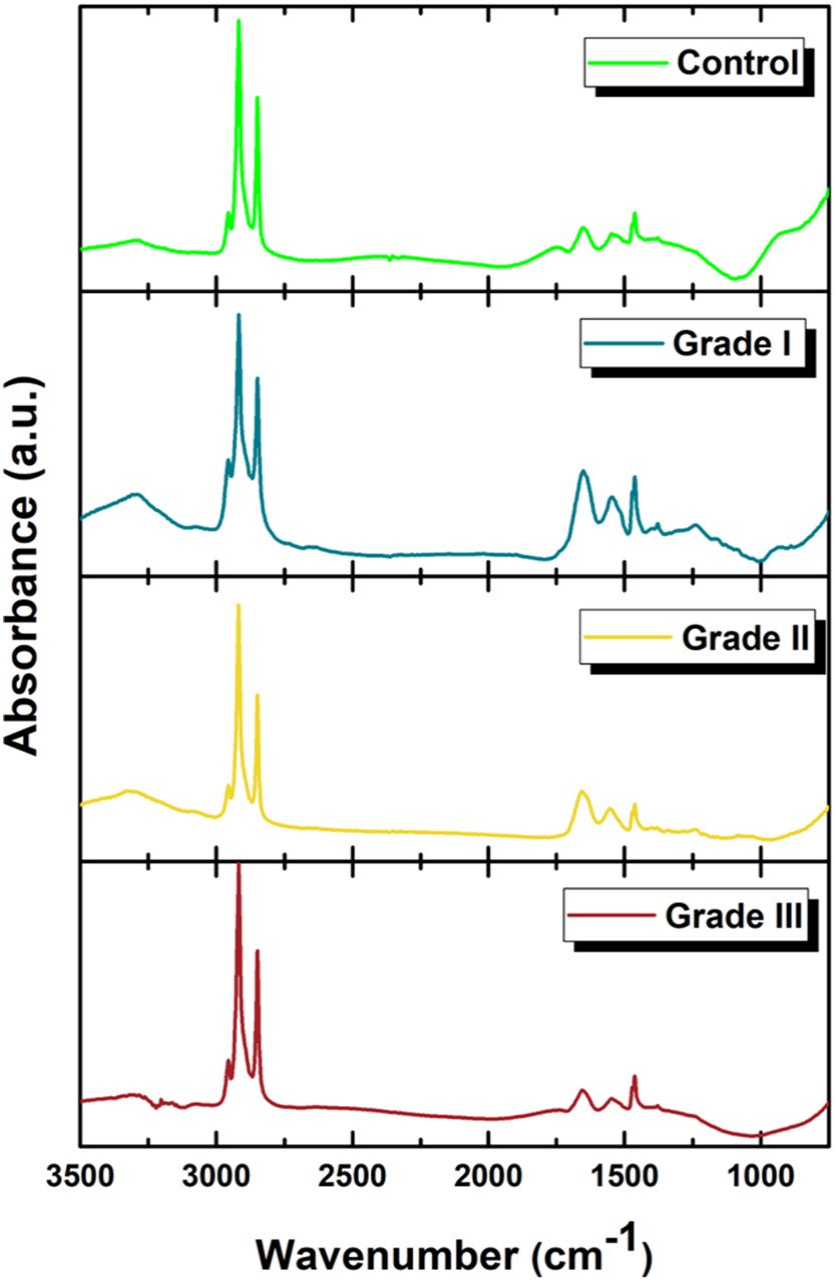
Stack graphs of characteristic FTIR spectra of breast tissue sections. Serial sections from those analyzed by THG microscopy in benign and grades I, II and III biopsies were used for the acquisition of FTIR spectra.

**Figure 7:**
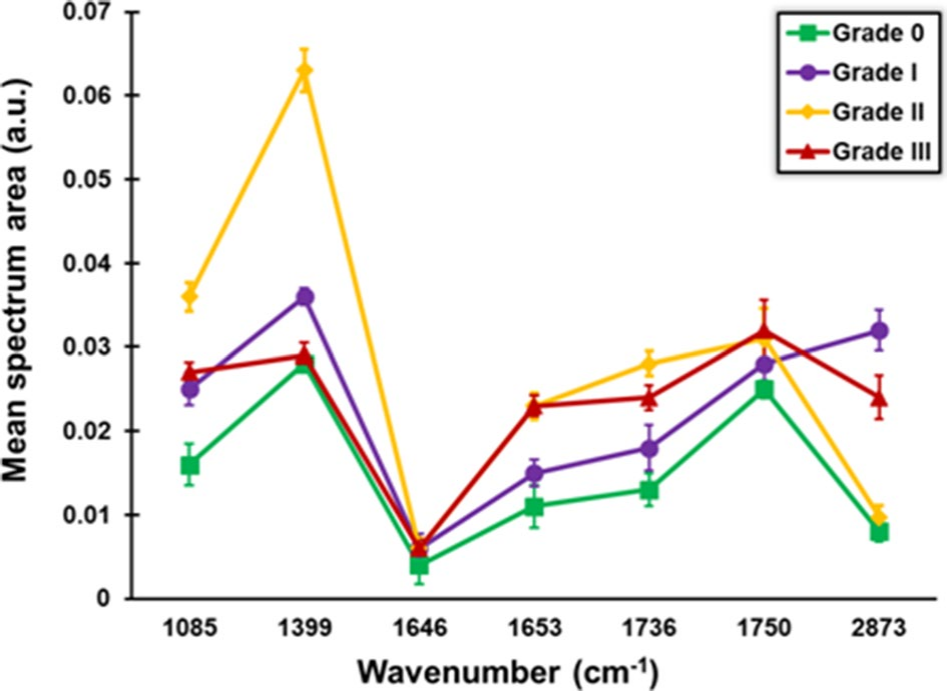
Concentration of lipid and phospholipid bonds in breast tissue biopsies. Data represent mean values of N = 15 areas for each case. Samples from two patients for each category were analyzed. Peaks 1,469 cm^−1^, 2,848 cm^−1^ and 2,916 cm^−1^ were omitted from the graph due to their higher values. Error bars indicate SEM.

Recent results had shown that THG signal detection could be correlated to specific chemical information provided by FTIR analysis in single cell specimens^18^. In order to evaluate whether such analysis could also apply to tissue sections, the samples studied in the present study were also submitted to FTIR analysis. Thus, serial sections from those analyzed by THG microscopy, with the same thickness (~ 5 μm) were used for the acquisition of FTIR spectra, by a Bruker HYPERION 2000 FT-IR microscope attached to a Bruker Vertex 70V FTIR spectrometer. Using such configuration, it became possible to focus and acquire spectra from specific areas of the samples (Fig. 6). Previous studies have shown that the increased THG signals in unstained tissue samples mostly arise from lipid bodies^19^, since they also co-localize with fluorescent staining of LBs in a plethora of biological specimens^6,18^. Thus, it could be postulated that the LB content of cells in the tissue contribute to the increased THG signal observed along with tumor progression. Therefore, FTIR analysis was focused on the spectra of lipid areas, while spectra integration allowed signal quantification (Table 2). The results showed that cancer tissues displayed higher concentration of lipid compounds at 1,085 cm^−1^, 1,399 cm^−1^, 1,469 cm^−1^, 1,646 cm^−1^, 1,653 cm^−1^, 1,736 cm^−1^, 1,750 cm^−1^, 2,848 cm^−1^, 2,873 cm^−1^ and 2,916 cm^−1^ (Fig. 7). Among the absorption wavelengths, the ones that correspond to cholesterol, phospholipids and C=O stretching modes mostly define lipid rafts and characterize activated cells^41^. The increased spectrum area in grade II tissues at 1,399 cm^−1^ could reflect the differential need in lipid nature of cancerous cells for migration to the axillary or sentinel lymph nodes.

**Table 2:** Integrated FTIR spectra.

Wavenumber (cm^−1^)	Description	Grade 0	Grade I	Grade II	Grade III
1,085	Mainly from membrane phospholipids (amide III)	0.016	0.025	0.036	0.027
1,399	CH_2_ wagging vibration of the acyl chains (phospholipids)	0.028	0.036	0.063	0.029
1,469	CH_2_ bending of the acyl chains of lipids	0.322	0.529	0.540	0.550
1646	C=O stretching (lipids)	0.004	0.006	0.006	0.006
1653	C=O stretching (lipids)	0.011	0.015	0.023	0.023
1736	C=O stretching (lipids)	0.013	0.018	0.028	0.024
1,750	C=O stretching (lipids)	0.025	0.028	0.031	0.032
2,848	Stretching vibrations of CH_2_ and CH_3_ of phospholipids, cholesterol	7.903	9.952	11.270	10.250
2,873	Symmetric stretching vibration of CH_3_ of acyl chains (lipids)	0.008	0.032	0.0097	0.024
2,916	Stretching vibrations of CH_2_ and CH_3_ of phospholipids, cholesterol	15.441	18.979	23.237	17.992

The integrated spectra peak area depicts the concentration of chemical bonds of lipids for Grade 0, Grade I, Grade II and Grade III tissue samples.

**Figure 8:**
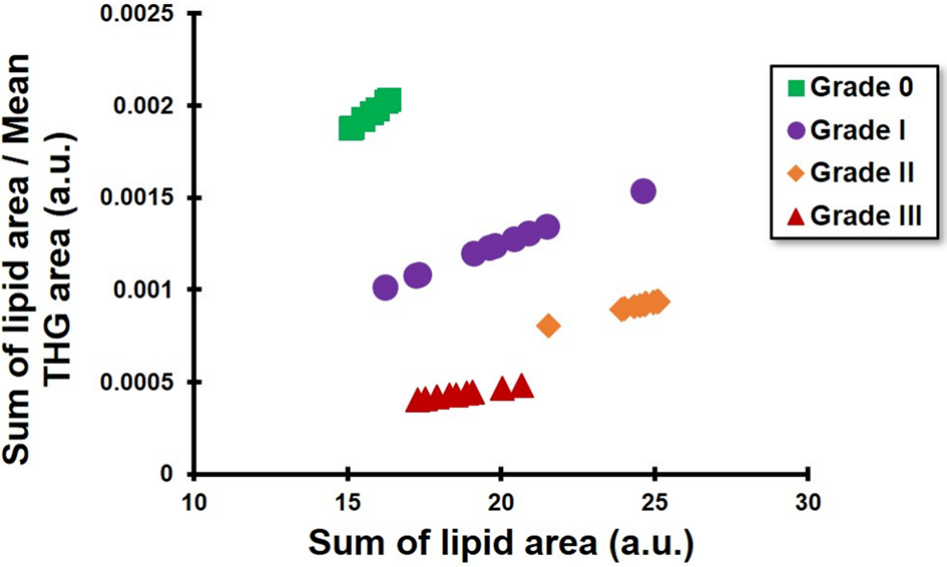
Scatter chart depicting sum of the lipid bond concentration divided with mean THG area of the corresponding breast tissues (y axis) as a function of the sum of lipid bond concentration (x axis). For this analysis N = 10 representative different tissue areas were examined.

Many studies demonstrate the need of cancer cells for large amounts of lipids, due to the generation of membranes during proliferation^42^. Quantification of the THG signal area, which mainly corresponds to LBs and inhomogeneities, could differentiate benign from malignant tissues and correlate THG signal area to tumor progression. In addition, FTIR analysis showed increased lipid distribution in malignant tissues. In an attempt to obtain complementary information concerning the THG quantification analysis in regard to lipids, FTIR spectra were correlated with the THG signal mean area. Concentrating on the FTIR spectra related to specific lipid regions (namely peaks at 1,399 cm^−1^, 1,469 cm^−1^, 1,736 cm^−1^, 1,750 cm^−1^, 2,873 cm^−1^ and 2,916 cm^−1^) and plotting the sum of lipid areas divided by the THG area as a function of the sum of lipid area, it was possible to clearly distinguish among the different grades of cancerous tissues (Fig. 8). Indeed, linear regression fitness analysis provided slopes at 1 × 10^–4^, 6 × 10^–5^, 4 × 10^–5^ and 2 × 10^–5^ for grades 0, I, II and III, respectively.

Moreover, linear regression analysis showed a significant correlation between the mean THG signal area and the integrated FTIR spectra of lipid compounds (R^2^ ~ 0.81), and cell volume with the integrated FTIR spectra of lipid compounds (R^2^ ~ 0.87). Therefore, combining THG quantification to FTIR lipid spectra integrated values, distinction and staging of breast cancer, could be achieved.

## Discussion

Rapid diagnosis and grading during surgery for malignant tissue exclusion from breast is an important task to achieve not only for securing tumor excision but also for providing information as to lymph node contamination and removal as appropriate. Thus, in cancer diagnosis, there is a growing need for the development of multimodal imaging-based diagnostic tools to objectively evaluate morphological features with subcellular resolution and molecular compositions that are closely associated with malignancy. Following such perspective, non-linear optical microscopy was proved to be useful in cancer research, providing high‐resolution, nondestructive imaging of tumors. In an effort to differentiate cancerous from benign breast tissues, in the current study, non-linear optical imaging and FTIR spectroscopy measurements were effectively applied to breast tumor sections and succeeded to discriminate among the different grades of cancer severity.

Non-linear optical modalities such as SHG, THG and MPEF provided unique morphologically information of breast biopsies, including collagen distribution, intracellular structures and inhomogeneities, as well as autofluorescence, using a single laser beam. In benign tissues, cells followed collagen orientation, which was well structured with specific continuity, while the increasing cancer severity correlated with collagen disorientation, which is in agreement with previous studies^34–36^. In this study, it was noticed that MPEF could not demonstrate any differences between the benign and cancerous tissues. Analysis of tissue autofluorescence signal is quite complicated, as it depends on many components, including lipofuscin, FAD, NADH, collagen and elastin. However, many studies have shown that MPEF could be used to discriminate cancerous tissues by collecting fluorescence lifetimes^43,44^.

THG imaging also provided the opportunity to calculate the surface area, volume, and nucleus/cytoplasm ratio of tumor cells, which serve as criteria for malignancy identification^38^. Although MNA and MCA in grade 0 samples were higher as compared to grades I, II and III, the N/C ratio was lower. Indeed, that was an expected finding, since grade 0 tumors although non-invasive, do contain tumor cells, which however can be considered as benign non-activated cells with proportional sizes in nucleus and cytoplasm, the ratio of which defines the normal phenotype of these cells. The decreased MNA and MCA levels obtained with grade III specimens was also expected, since in this state most malignant cells are found in lymph nodes and not in the breast itself, displaying thus a characteristic type of morphology.

Quantification of the THG recorded signal area and volume, which mainly detect intracellular LB structures and inhomogeneities without the need of staining, allowed distinction between benign and malignant tissues. Such experimentation showed that cancer cells displayed a statistically significant increase of cell volume as compared to grade 0-derived cells that was also correlated with cancer severity for grades I, II and III. Normalization of the obtained results by dividing THG signal area of each cell to the respective cell volume reproduced the observed difference between benign and cancerous cells in the tissues tested. Such observation indicated that independently of the cell volume, a significant increase of the THG signal area characterized malignant cells. Similar results were obtained when THG signal intensity was evaluated, which was also shown to increase with cancer severity. The presented findings are in agreement with previous observations showing that THG modality could detect increased lipid profiles in inflammation or cancer^18,45^.

Complementary, FTIR spectra could be used to correlate specific chemical information in the different examined tissues. FTIR analysis showed an increased lipid content during cancer progression. Moreover, correlating the FTIR findings with the mean THG signal area, linear regression analysis showed a strong agreement with the increased LB profile during tumor progression, also allowing discrimination of the different grades.

Notably, these data are interrelated to those from clinical diagnostic approaches, since non-linear optical images were comparable to standard H&E staining images, also enabling the analysis of cell size, cell shape, nuclear shape and intracellular organelles without the need of a specific staining. Thus, the unique attributes of non-linear optical microscopy described herein provide promising imaging modalities for disease diagnostics in clinic*.* A limiting parameter in the application of the proposed label-free non-linear technology is the lack of specific chemical information. Although this is provided by other technologies, such as Coherent Anti-stokes Raman Scattering (CARS) and Stimulated Raman Scattering (SRS), they are much more expensive and technical challenging. At its current state, the depth of THG imaging detection is approximately 500 μm that limits its application to characterization of superficial cancer tissues during surgery. However, proper device development could further expand the applications of non-linear technology for use in the clinical practice.

## Conclusions

The method described herein enabled non-linear microscopy techniques to visualize and quantitatively differentiate malignant cells in benign and cancerous human breast biopsies, promising thus a label-free diagnosis, which could also be applied to fresh tissues during surgery. A big advantage of the proposed technology is its speed as a diagnostic tool. It offers fast—in the order of minutes—and reliable outputs in cancer diagnosis as it utilizes unstained tissue samples, without the need of any pre-processing, like for example the standard H&E staining method that lasts for several hours. Moreover, advances in microscopy with the generation of new modalities of image contrast, along with advances in deep learning algorithms for the automatic, quick identification of cancer cells in tissues/organs and categorization of the severity of the disease present a great opportunity for an objective diagnostic tool that will indicate molecular and structural changes for prognosis and treatment. New advances in imaging systems and multimodal optical contrast will continue widen the capacity for diagnosis of diseases, where instead of simple eye inspection, objective new tools could be developed for the early diagnosis of inflammation in various diseases.

## Materials and methods

### Non-linear microscopy setup

In this study, THG, SHG and MPEF microscopy techniques were combined to collect morphological information of tissue biopsies. The custom-made experimental setup used herein, allowed simultaneous collection of two different non-linear signals, and has been previously described in detail^18,45^. Briefly, the experimental setup consisted of an Amplitude systems t-pulse femtosecond laser (1,028 nm, 50 MHz, 1 W, 200 fs) and a modified Nikon upright microscope (Nikon Eclipse ME600D, Tokyo, Japan). The energy per pulse at the sample plane was 0.4nJ. A high numerical aperture objective lens (Carl Zeiss, C-Achroplan 32x, NA 0.85, water immersion) was employed for the tight focusing of the laser beam onto the sample. The scanning procedure (xy direction) was performed with a pair of galvanometric mirrors (Cambridge Tech. 6210H). The samples were fitted into a motorized xyz translation stage (Standa 8MT167-100, Vilinius, Lithuania) and the focal plane was selected with this stage (1 μm resolution). For wide scanning regions (~ mm) a synchronized movement of galvo mirrors with the xyz stage was performed. Lab View interface controlled both scanning and data acquisition procedures. SHG & MPEF signals were collected in the backward direction using a photomultiplier tube (PMT Hamamatsu R4220, Hamamatsu city, Japan). The photomultiplier tube was attached at the position of the microscope eye-piece. A bandpass interference filter (CVI 514 nm) was placed at the PMT input to cut off the reflected laser light and solely detected SHG signals from the samples. The short bandwidth of the filter (3 nm) used herein, verified that only a minimal amount of fluorescence signals can be detected as an extremely weak constant background. In case of MPEF, a short pass filter (SPF 700 nm, CVI Laser, Albuquerque, New Mexico) and a notch filter (NF 514-17 Thorlabs, Germany) were placed at the PMT input for the detection of fluorescence signals from the samples. SHG and MPEF signals were recorded in distinct set of measurements. THG signals were detected simultaneously in the forward direction, by employing a colored glass filter (U 340 nm Hoya, UOG optics, Cambridge, UK) and a second PMT (Hamamatsu H9305-04, Hamamatsu city, Japan). Typical time duration for obtaining a 2-D 500 × 500 pixels non-linear image is one (1) second. For large scanning areas, a 2D 1 × 1 mm image could be acquired in 100 s. To improve the signal to noise ratio (SNR), 20 scans were realized for each sample plane. A series of 2D optical sections were obtained at 1 μm intervals (z stack) and projected (maximum intensity projection) onto a single plane. The depth of scanning was dependent of the tissue width (~ 10 slices covered the whole sample). Image J software was used for data viewing and processing (NIH, https://imagej.nih.gov/ij/).

### FTIR micro-spectroscopy

As previously described by our laboratory^18^, tissues were deposited on IR transparent BaF_2_ windows (20 mm dia × 2 mm Crystran optical windows). Transmission measurements were performed using a Bruker Vertex 70v FT-IR spectrometer attached to a Bruker HYPERION 2000 FT-IR microscope by means of a 15 × magnification objective lens and a numerical aperture (ΝΑ) of 0.4, a KBr beamsplitter, and a liquid nitrogen (LN) cooled mercury cadmium telluride (MCT) detector. In all cases, interferograms were collected in a spectral range of 7,500–380 cm^−1^, at 4 cm^−1^ resolution (32 scans), apodized with a 3-Term Blackman-Harris function, and Fourier transformed with two levels of zero filling in order to yield spectra encoded at 2 cm^−1^ interval. Before scanning, BaF_2_ background was recorded and each sample spectrum was obtained by automatic subtraction of the background.

### Spectra analysis

Following the previously defined parameters^18^, the FTIR spectra were analyzed/processed using OPUS software supplied by the instrument manufacturer (OPUS software package; Bruker Optik GmbH, Germany). The FTIR spectra along with their first and second derivative curves were used in order to highlight the components of different spectral regions and to determine the approximate values of the peak positions of the components. The integrated area of the recorded FTIR peaks were determined using OPUS software in order to estimate the concentration of the corresponding bonds.

### Biological samples

Benign (control) and cancerous tissues were obtained from a total of 15 patients, including two cases of benign bearing tumor (grade 0, ductal carcinoma), four patients with grade I tumor, five patients with grade II tumor and four patients with grade III tumor. All patients were older than 60 years of age and had provided signed informed consent to donate the spare biopsy tissues to research. The experimental protocol was approved by the Ethics and Scientific Committees of the University Hospital of Heraklion (IDs 16/16-10-2019 (579) and 4428/10-07-2019; Crete, Greece). All methods were carried out in accordance with relevant guidelines and regulations.

The breast cancer grades considered in this work have followed the analysis described by Akram et al.^46^, as conveyed in Table 3.

**Table 3:** Features of breast cancer tissue grading.

Tumor grade	Features
Grade 0	Non-invasive and non-cancerous cells in breast
Grade I	1A (tumor > 2 cm) no lymph node invasion
	1B (tumor > 0.2 cm) in lymph node
Grade II	2A in axillary lymph node or sentinel lymph node
	2B (tumor > 5 cm) cannot reach axillary lymph
Grade III	3A any size/4–9 axillary lymph nodes or sentinel lymph nodes
	3B any size/up to 9 axillary lymph nodes or to sentinel lymph nodes
	3C spread tumor > 10 axillary lymph nodes
Grade IV	Advanced metastatic with spreading to other organs

Tissue samples were fixed in formalin to cross link proteins and 1% osmium tetroxide to immobilize lipids^33^, embedded in paraffin, sliced in 5–10 μm-thick histological sections and routinely stained with hematoxylin and eosin (H&E) for optical microscopy examination^47^. For non-linear imaging, unstained tissue slices were placed on special very thin (0.07 mm) round glass microscope slides of 3.5 cm diameter to provide a flat sample surface. Moreover, H&E images of serial sections of the same sample were recorded for evaluation and comparison with the non-linear data. Tissue sections were in some cases stained with nile red (Sigma-Aldrich Co., MO, USA) according to the manufacturer’s instructions for the detection of lipid bodies.

### Data analysis

**Figure 9:**
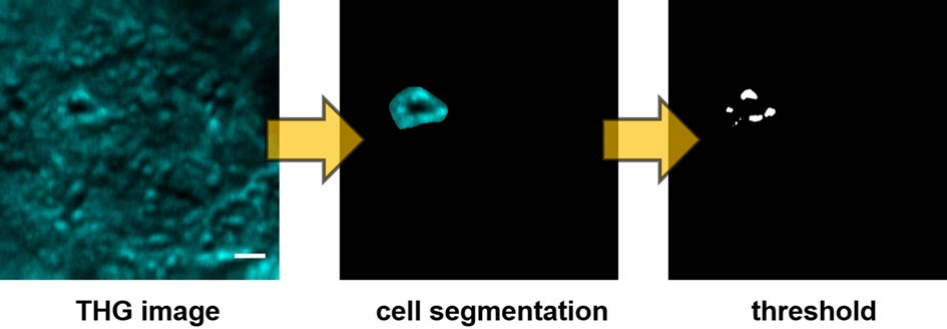
Illustration of THG tissue processing. Manual isolation of individual cells for quantification analysis (based on LB content, inhomogeneities and membranes).

For the identification of the cells in the tissue (cell segmentation), a scanning region of 45 × 45 μm^2^ was selected from a larger field of view (Supplement Fig. 2), and images were collected from the whole tissue volume of the regions (3-D images). This scanning region was selected to identify the cells and study the intracellular structures. This selection was assisted and verified with the white light observation of the same investigated area and the information derived from the sequential H&E slice images of the tissue. The cells could be identified through the 3D dimension of the tissue. In particular, 3D obtained THG images revealed the margins between the cells due to the inhomogeneities (membranes) as well as the black central area corresponding to the nucleus. These observations are in perfect agreement with our previous studies employing the same experimental apparatus and irradiation conditions to breast cancer cell lines and mouse T-cells^18,45^. After optically isolating the cells from each sample, these were submitted to quantification processing (Fig. 9), including the calculation of each cell volume, the mean THG area of each cell and the mean THG intensity signal of the tissue area. The volume of each cell was calculated by measuring the surface of the cell of each slice and then multiplying by the number of slices of the tissue that was evaluated.

Quantification analysis was performed as previously described^45^. Briefly, working under constant irradiation conditions (mean energy per pulse, linear polarization of the incident beam at the sample plane, dimensions of the scanning region, number of pixels, amplification of the PMT units) for all samples, THG intensity values were collected from the photomultipliers’ tubes (PMTs), stored in 2D 500 × 500 matrices that represented a single slice image of the sample. Image J software was used for image processing and thresholding. THG signal quantification was performed by setting a constant global threshold for all the investigated tissues, in the obtained normalized 8-bit slice images, so that regions generating high levels of non-linear signal were solely detected and isolated. The threshold was set in a way to allow the higher THG signal that indicates mainly inhomogeneities and lipid content of the cell area to be analyzed and measured. Consequently, the obtained calculated area of each section corresponded to lipid bodies and discontinuities in the tissue. The quantification of mean THG area was calculated from all the sequential z slices of each tissue. Forty different areas in the tissue were tested in each case.

The THG intensity quantification was performed as previously described from our laboratory^18^. Briefly, the values of a number of N samples were normalized to their maximum THG intensity value for each 2D tissue section. Quantification analysis was accomplished by setting a second threshold based on a constant PMT value, so that only the regions that provide high THG signals could be examined. An algorithm that was designed and programmed in MATLAB environment was employed for the estimation of mean pixel value. The integrated THG intensity over total pixel area for each section of the tissue was calculated by multiplying the representative area with the mean intensity value of the corresponding pixels. The weighted mean pixel value of each tissue was obtained by repeating this procedure for all the sections of the sample. The difference between the various samples was assessed by repeated ANOVA measures. Statistics were performed using GraphPad Prism 6.01 (Graphpad Software, La Jolla, California).

## Supplementary information

Supplementary file1
