# Supplementary material for: Third Harmonic Generation microscopy distinguishes malignant cell grade in human breast tissue biopsies
Source: Sci Rep. 2020 Jul 6;10:11055. doi: 10.1038/s41598-020-67857-y (PMC7338369; doi:10.1038/s41598-020-67857-y)
Supplement: Supplementary file 1 — Supplementary file1 [file 41598_2020_67857_MOESM1_ESM.pdf]

Supplementary

Supplement Figure 1

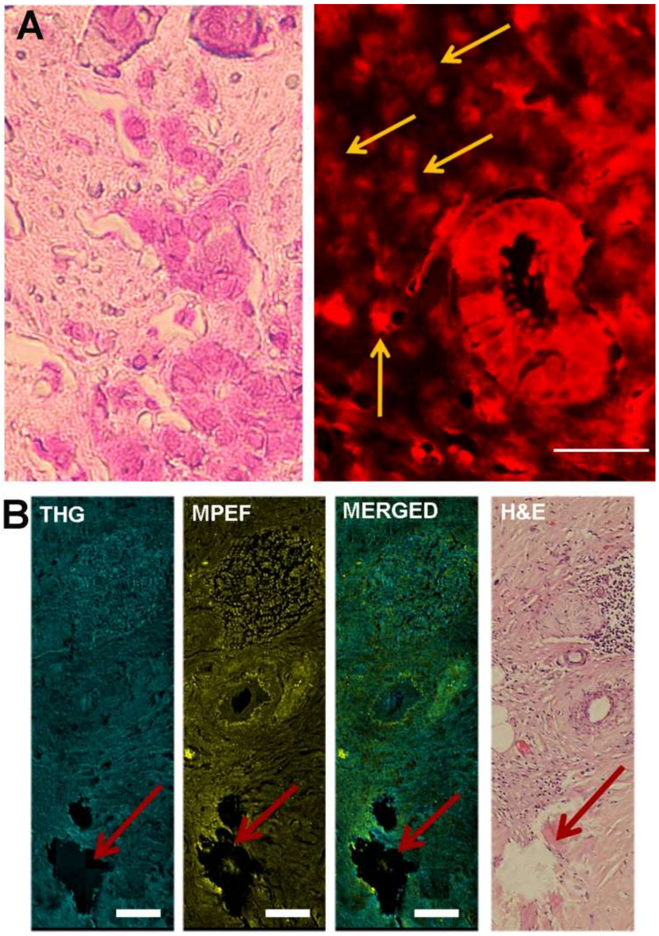

Supplement Figure 1: Serial sections of paraffin breast tissue were stained with H&E (A left) or nile red (A right) as described in Methods (H&E staining is not compatible with nile red staining[47], therefore serial sections had to be used in the procedures). Arrows indicate LB staining. THG, MPEF and merged imaging (B upper images) as well as H&E staining discriminate empty spaces (red arrows). Scale bar depicts 100 µm.

Supplement Figure 2

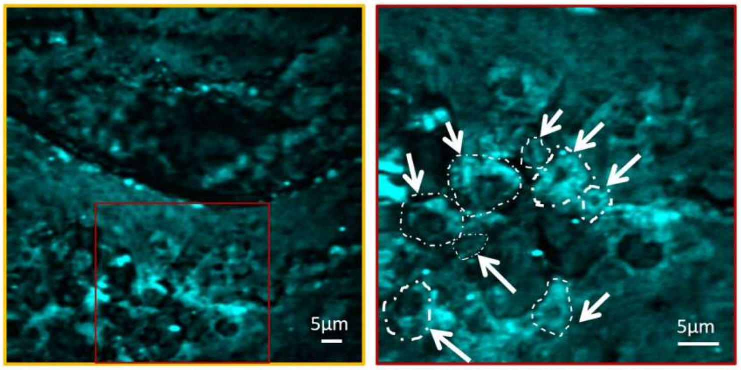

Supplement Figure 2: 2D THG images of different field of views (FOV) left image 90 µm^2 and right image 45 µm^2 of breast cancer grade II. Red square represents the area that is zoomed in the right picture. White arrows in the right picture indicate some of the cells that were manually segmented.
